# Clinical study on the feasibility of new thrombus markers in predicting massive cerebral infarction

**DOI:** 10.3389/fneur.2022.942887

**Published:** 2023-01-25

**Authors:** Xiaoxia Zhao, Siyu Yang, Ruining Lei, Qiaoyan Duan, Jundong Li, Jiangtao Meng, Lei Sun

**Affiliations:** ^1^Department of Neurology, Shanxi Provincial People's Hospital, Taiyuan, Shanxi, China; ^2^Department of Neurology, Fifth Hospital of Shanxi Medical University, Taiyuan, Shanxi, China; ^3^Clinical Laboratory, Shanxi Provincial People's Hospital, Taiyuan, Shanxi, China; ^4^Medical Imaging Department, Shanxi Provincial People's Hospital, Taiyuan, Shanxi, China; ^5^Zhao Furun Famous Doctor Studio in Shanxi Province, Taiyuan, Shanxi, China

**Keywords:** thrombin-antithrombin complex, plasmin-α2 plasmin inhibitor complex, tissue prothrombin activator-prothrombin activator inhibitor complex, thrombomodulin, massive cerebral infarction, diagnosis

## Abstract

**Objective:**

This study investigated the diagnostic performance of the thrombin-antithrombin complex (TAT), plasmin-α2 plasmin inhibitor complex (PIC), tissue plasminogen activator-plasminogen activator inhibitor complex (t-PAIC), and thrombomodulin (TM) in the early identification of massive cerebral infarction.

**Method:**

A total of 423 patients with cerebral infarction confirmed by imaging examination were divided into the massive cerebral infarction (MCI) group and the non-massive cerebral infarction (NMCI) group. TAT, PIC, t-PAIC, and TM were measured immediately after admission. The diagnostic performance was analyzed by the receiver characteristic operating curve (ROC).

**Result:**

The median plasma concentrations of TAT, PIC, and t-PAIC in patients with MCI at early onset were 5.10 ng/ml, 1.11 μg/ml, and 8.80 ng/ml, respectively, which were higher than those in patients with NMCI (2.20 ng/ml, 0.59 μg/ml, and 7.35 ng/ml), and the difference was statistically significant (*P* < 0.001). TAT was shown to be an independent risk factor for the development of massive cerebral infarction by a multivariate logistic regression analysis (OR = 1.138). A ROC curve analysis showed that PIC had the best performance in identifying MCI at an early stage (AUC = 82.8%), with a sensitivity of 80.7% and a specificity of 76.2% when the PIC concentration was ≥0.8 μg/ml; TAT had the highest specificity in identifying MCI, with a specificity of 80.6% when the TAT concentration was ≥3.97 ng/ml.

**Conclusion:**

The detection of PIC, TAT, t-PAIC, and TM is a comprehensive assessment of vascular endothelial damage and activation of the coagulation and fibrinolytic systems and has diagnostic value for early identification of patients with MCI, which, together with its ease of detection, can be used as a plasma marker for early identification of large vessel occlusion.

## 1. Introduction

Massive cerebral infarction (MCI) is caused by internal carotid or middle cerebral artery trunk occlusion, with a mortality rate of 53–78% (1). Early diagnosis before admission and transportation to advanced stroke centers for timely thrombolysis or embolectomy treatment is significant to reduce the burden of serious consequences. Although imaging examination is a reliable method to determine vascular occlusion, it is usually not possible to perform it before admission, so it is very critical to find appropriate plasma markers for early identification of massive cerebral infarction.

Ischemic stroke is mostly caused by intravascular thrombosis due to coagulation abnormalities, and the occurrence of ischemic stroke will cause changes in the coagulation and fibrinolytic system; the two interact with each other (2). Previous studies have shown that the plasma concentrations of new thrombus markers are related to the occurrence and development of cardiovascular and cerebrovascular diseases closely. Thrombomodulin (TM) has been confirmed to be involved in the regulation of the body's coagulation and fibrinolytic system (3), and it has been found that the TM content in the plasma is associated with vascular endothelial injurious diseases (4–6). Plasmin-α2 antiplasmin complex (PIC) can reflect the activation of the fibrinolytic system and is more sensitive than D-dimer and fibrinogen degradation products (FDP) (7). Thrombin-antithrombin complex (TAT) is a sensitive marker of thrombin formation and has a certain predictive role in a variety of ischemic diseases (8–10). The high concentration of tissue plasminogen activator-plasminogen activator inhibitor complex (t-PAIC) in the blood indicates damage to the fibrinolytic system and can be used as an independent predictor of cardiovascular mortality (11). These new thrombus markers can be detected by a high-sensitivity chemiluminescence system rapidly and accurately, and the results can be obtained within 17 min (12), offering the possibility of early identification of large vessel occlusions.

To investigate whether the plasma concentrations of novel thrombus markers (TM, PAP, TAT, and t-PAIC) are associated with cerebral infarct size, we measured the plasma concentrations of these thrombus markers in patients with cerebral infarction, assessed their diagnostic performance for massive cerebral infarction for prehospital identification of macrovascular occlusion, and laid the foundation for further exploration of the molecular mechanism of massive cerebral infarction.

## 2. Materials and methods

### 2.1. Patients

A total of 423 patients (118 female patients and 305 male patients) with acute ischemic stroke, including 57 patients with massive cerebral infarction, were enrolled in the study from July 2019 to March 2022. The study protocol was reviewed and approved by the Human Ethical Review Committee of Shanxi Provincial People's Hospital. Written informed consent was obtained from the patient or close relatives.

The inclusion criteria were as follows: (1) aged ≥18 years; (2) admitted within 24 h after the onset of acute cerebral infarction; (3) acute cerebral infarction was defined as a rapidly progressive neurological deficit of vascular origin lasting, diagnosed as ischemic stroke by head CT or MRI examination (13); and (4) informed consent.

The exclusion criteria were as follows: (1) traumatic brain injury within the past 1 week; (2) the patient was discharged or transferred due to an unknown prognosis during the observation period (7 days after onset); (3) patients with malignant tumors of any organ or system; (4) patients with severe lung, liver, kidney, blood coagulation system, immune system, and other important organs or systems injury; (5) patients with lower limb thrombosis, pulmonary embolism, and other serious thrombosis diseases affecting coagulation function and injuries using anticoagulant drugs; (6) patients with severe neurological disability (modified Rankin Scale score, mRS ≥2 points) before onset; and (7) patients with cerebral hemorrhage, brainstem infarction, or bilateral cerebral hemispheric infarction.

### 2.2. Baseline indicator

Patient age, gender, and risk factors for cerebral infarction (hypertension, diabetes, atrial fibrillation, smoking history, and alcohol history) were recorded.

### 2.3. Imaging examination

After completing magnetic resonance imaging on admission, two senior imaging physicians who ignored the results of plasma tests independently classified the patients into the massive cerebral infarction (MCI) group and the non-massive cerebral infarction (NMCI) group according to the results of the imaging examinations, and in case of disagreement, the decision was made by mutual discussion between the two.

### 2.4. Blood sampling and assay procedure

Immediately after admission, 2.7 ml of cubital venous blood was collected at one time and injected into BD vacuum blood collection tubes containing 0.3 ml of 0.109 mol/L sodium citrate anticoagulant (9:1 ratio), inverted, and mixed well. After centrifugation at 3,500 r/min for 15 min, platelet-poor plasma was obtained. These plasma samples were all quoted in Eppendorf tubes and then stored at −80°C. The assay was completed within 24 h.

TAT, TM, t-PAIC, and PIC were determined by chemiluminescent enzyme immunoassay (instrument: Sysmex HISCL-5,000 automatic chemiluminescence analyzer and its supporting chemiluminescent enzyme immunoassay reagent). The samples were mixed with reagents for incubation, and then the immune reaction and chemiluminescent enzyme reaction occurred in turn. Using the high specificity of the antigen-antibody reaction and the high sensitivity of luminescence analysis, the content of the analyte in the sample was determined by counting the photons emitted in the enzyme reaction quickly and automatically.

### 2.5. Criteria for MCI

MCI was defined as the Alberta Stroke Program Early CT Score (ASPECTS) < 6 on CT or magnetic resonance diffusion imaging (DWI) images (14), infarct volume ≥70 ml (15), or infarct size ≥2/3 middle cerebral artery territory (16). ASPECTS scoring method: The blood supply area of the middle cerebral artery was divided into 10 regions, i.e., M1–M6, as well as the nucleus accumbens, caudate nucleus, internal capsule, and insula; each region was scored as 1 point, totaling 10 points, and 1 point was subtracted for the presence of 1 EIC region; an ASPECT score of 10 means no early ischemic changes, while a score of 0 indicates the presence of extensive ischemic foci in brain tissue (17).

### 2.6. Data statistics

Normal distribution continuous variables were expressed as mean ± standard deviation (SD) and compared using the independent sample *t*-test; non normal distribution continuous variables were expressed as median (interquartile range) and compared using the Mann-Whitney U test. Categorical variables were presented as numbers and percentages and compared using the chi-square test. Items with a *P* ≤ 0.05 in the univariate analysis were included in the multivariate logistic regression analysis to assess the risk factors for the occurrence of massive cerebral infarction. The receiver operating characteristic curve (ROC) was used to analyze the accuracy of different plasma markers in the diagnosis of MCI, and the Youden index was calculated, where the best cut-off value was obtained when the Youden index was maximum. The area under the ROC curve (AUC) of 70–90% suggested that it was acceptable and had good accuracy. A *p* ≤ 0.05 was considered statistically significant. SPSS statistical software, version 25.0 (IBM, Armonk, NY, USA), was used for all statistical analyses.

## 3. Results

### 3.1. Baseline data analysis

A total of 423 patients with cerebral infarction were included in the study from July 2019 to March 2022, and 57 patients (13.5%) were diagnosed with massive cerebral infarction by imaging examination.

Differences in gender, age, and risk factors for cerebral infarction such as atrial fibrillation, hypertension, smoking history of diabetes, and drinking history between the MCI group and the NMCI group are not statistically significant (Table 1).

**Table 1:** Comparison of baseline data between the MCI group and the NMCI group.

**Characters**		**NMCI (*n* = 366)**	**MCI (*n* = 57)**	**χ2/Z**	***P*-value**
Gender	Male	265 (72.4%)	40 (70.2%)	0.122	0.727
	Female	101 (27.6%)	17 (29.8%)		
Age (years)		64.0 (54.0, 72.0)	64.5 (53.0, 72.5)	−0.098	0.922
Risk factors	Hypertension	240 (65.6%)	37 (64.9%)	0.010	0.922
	Diabetes	118 (32.2%)	18 (31.6%)	0.010	0.921
	Atrial fibrillation	32 (8.7%)	9 (15.8%)	2.798	0.094
	Smoking	225 (61.5%)	32 (56.1%)	0.589	0.443
	Alcohol	155(42.3%)	31 (54.4%)	2.900	0.089

### 3.2. Plasma concentrations of TM, PIC, TAT, and t-PAIC in different groups

**Figure 1:**
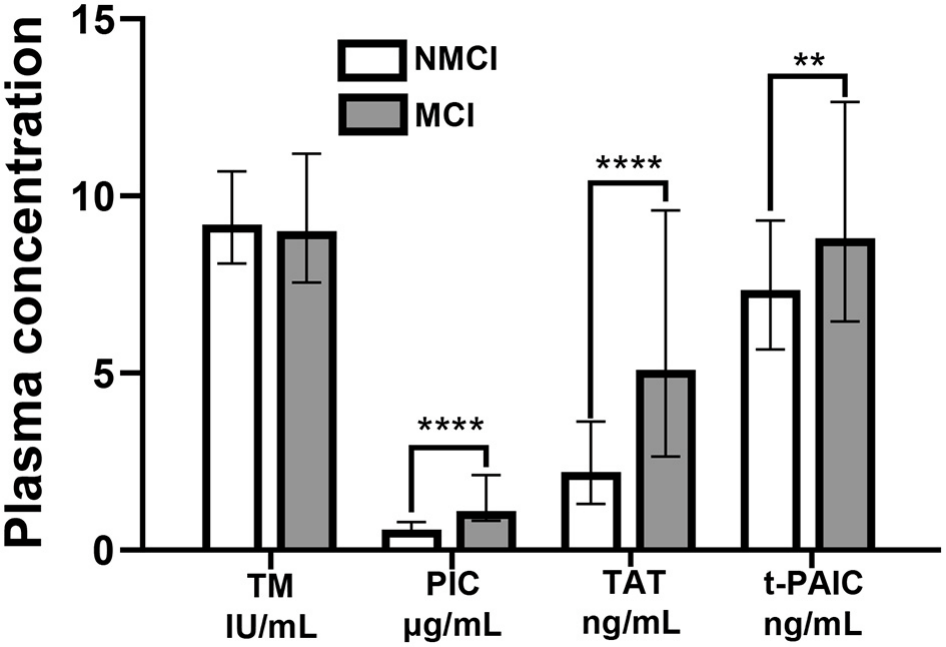
Plasma concentrations of TM, PIC, TAT, and t-PAIC in the MCI group and the NMCI group. **P < 0.01, ****P < 0.0001.

The plasma concentrations of PIC, TAT, and t-PAIC in the MCI group were higher than those in the NMCI group, and the difference between the two groups was statistically significant (Z = −7.978, *P* < 0.001; Z = – 6.621, *P* < 0.001; Z = −2.709, *P* = 0.007). There was no significant difference in TM plasma concentrations between the two groups (Z = −0.119, *P* = 0.905) (Figure 1).

### 3.3. Logistic regression analysis of risk factors for massive cerebral infarction

Differences in PIC, TAT, and t-PAIC between the two groups were statistically significant. A multivariable logistic regression analysis showed that the high concentration of TAT plasma was statistically significant and associated with the occurrence of MCI independently (Table 2). When the concentration of plasma TAT in patients with cerebral infarction increased by 1 ng/ml, the probability of MCI increased by 1.138 times (95% CI:1.065, 1.215; *P* < 0.001).

**Table 2:** Logistic regression analysis of risk factors for MCI.

**Variable**	**OR (95%CI)**	***P*-value**
PIC	1.037 (0.986–1.091)	0.162
TAT	1.138 (1.065–1.215)	< 0.001
t-PAIC	1.034 (0.969–1.103)	0.307

### 3.4. Diagnostic value of plasma PIC, TAT, and t-PAIC concentrations in massive cerebral infarction

**Figure 2:**
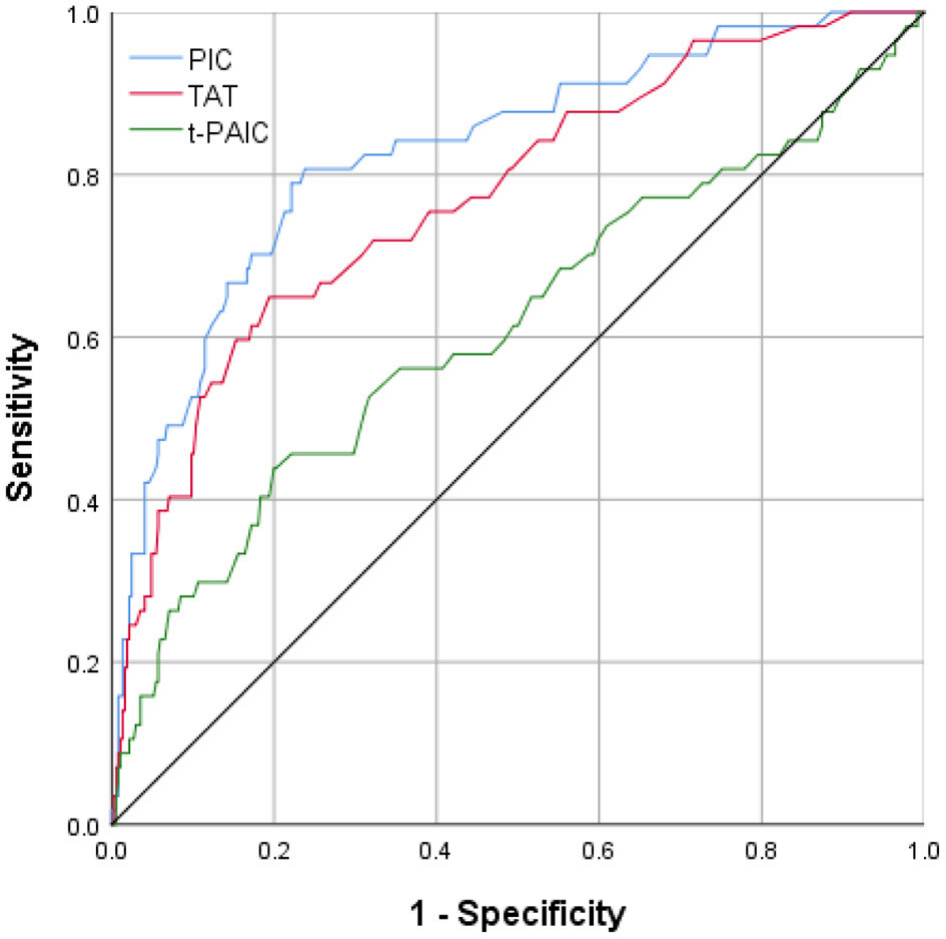
The area under ROC curve of the levels of plasma PIC, TAT, and t-PAIC in patients with AIS prediction of MCI.

All three novel thrombus markers showed good accuracy (>80%) for the diagnosis of MCI by the Hosmer-Lemeshow goodness-of-fit test analysis (Table 3). By a ROC curve analysis (Figure 2), both PIC and TAT showed good diagnostic performance for MCI (AUC ≥ 70%). PIC showed the best diagnostic performance (AUC = 82.8%) when the plasma concentration of PIC was ≥0.80 μg/ml, the maximum Youden index of 0.569 was obtained, the sensitivity and specificity reached 80.7 and 76.2%, respectively, and the sensitivity was the best among the three markers. The highest specificity of TAT for the diagnosis of MCI was obtained when the TAT concentration in plasma was ≥3.97 ng/ml, with a maximum Youden index of 0.455, a specificity of up to 80.6%, and a poor sensitivity of 64.9%.

**Table 3:** Performance analysis of three markers for diagnosing MCI.

**Classifier**	**Calibration**	**Discrimination**	**95% CI**	**Cut-off value**	**Sensitivity**	**Specificity**
PIC	86.5%	82.8%	76.8–88.9%	0.80 ng/mL	80.7%	76.2%
TAT	87.0%	77.2%	70.4–84.0%	3.97 ng/ml	64.9%	80.6%
t-PAIC	86.1%	61.1%	52.4–69.9%	9.85 ng/m	43.9%	80.1%

## 4. Discussion

In this study, we examined the plasma concentrations of novel thrombotic markers in 423 patients with cerebral infarction immediately after admission within 24 h after onset, investigated the correlation between four novel thrombotic markers (TM, PIC, TAT, and t-PAIC) and the occurrence of MCI, and assessed their diagnostic performance for the occurrence of MCI. We found that compared with patients with NMCI, patients with MCI had increased plasma PIC, TAT, and t-PAIC concentrations, which could assist in the identification of MCI in the early stage of cerebral infarction and provide a reference for prehospital identification of macrovascular occlusion.

PIC is the main enzyme that dissolves fibrin *in vivo*, plasmin. In the absence of fibrin, it combines with its representative inhibitor, α2-antiplasmin (α2-PI), 1:1 to form a stable complex (18), which has a half-life of ~6 h (19) and is easier to measure than plasmin with a half-life of only a few seconds; and its plasma concentration is not affected by antiplatelet and anticoagulant drugs, nor does it affect the production of plasmin, so it can represent the degree of plasmin activation more accurately (20). In this study, we found that the plasma PIC concentration in patients with MCI was higher than that in patients with NMCI (*P* < 0.001), suggesting that the degree of activation of the fibrinolytic system is related to the infarct size, and the fibrinolytic system is more hyperactive in patients with MCI, which is one of the reasons why patients with MCI are more likely to have hemorrhagic transformation. This result is similar to the findings of Sato et al. further confirming that the plasma PIC concentration can reflect the severity of cerebral infarction (21). In addition, a ROC curve analysis revealed that PIC had a high diagnostic value for the occurrence of massive infarction, with an AUC of 82.8% (95% CI: 76.8, 88.9%), and when the plasma concentration of PIC was ≥0.80 μg/ml, the sensitivity and specificity reached 80.7 and 76.2%, respectively. We did not find that high plasma PIC concentration was an independent risk factor for the occurrence of MCI using a multivariable logistic regression analysis, which may be related to the fact that the concentration of PIC in plasma is affected by other cerebrovascular disease risk factors such as atrial fibrillation and atherosclerosis (21, 22).

TAT is produced during the formation of fibrin polymers and is a complex formed by the combination of thrombin and antithrombin in the body. The increase in TAT indicates that thrombin is being generated while antithrombin is continuously consumed. Elevated TAT concentrations have been found in diseases affecting the coagulation system, such as venous thrombosis, malignant tumors, and disseminated intravascular coagulation (DIC), suggesting that the body is in a hypercoagulable state (23–25). Moreover, TAT is upstream of traditional coagulation test items such as D-dimer and FDA and has been confirmed to be more sensitive in reflecting the body's coagulation activation (7). By comparing the plasma TAT concentration between the two groups, we found that the plasma TAT concentration was significantly higher in patients with MCI, and TAT was found to be one of the independent risk factors for the occurrence of massive cerebral infarction by a logistic multivariate regression analysis. Excessive activation of the coagulation system may be one of the mechanisms for the occurrence and development of massive cerebral infarction, or the body may change after the occurrence of massive cerebral infarction. Large-sample prospective studies are needed to explore their causal relationship. The diagnostic performance of TAT for MCI was analyzed by further ROC curve analysis, and it was found that TAT could diagnose the occurrence of MCI and reflect the occurrence of MCI and NMCI accurately, reflecting the possibility of macrovascular occlusion (AUC = 87.0%, 95% CI: 64.9, 80.6%). At the same time, the high concentration of plasma TAT in patients with MCI suggests that the occurrence and development of massive cerebral infarction are accompanied by excessive activation of thrombin, and the increase of TAT suggests the necessity of initiating anticoagulation after the occurrence of MCI. However, patients with MCI are often accompanied by hyperfibrinolysis in the early stages, and the anticoagulation regimen and the time to initiate anticoagulant therapy require further study.

t-PAIC is a complex formed by the combination of tissue-type plasminogen activator (t-PA) and physiological inhibitory factor plasminogen activator inhibitor-1 (PAI-1). t-PA is a key factor in the exogenous activation pathway of the fibrinolytic system *in vivo*, while PAI-1 can specifically bind t-PA to form t-PAIC to interfere with the activation of plasmin. The increased plasma t-PAIC concentration reflects the impairment of the activation pathway in patients (26). In addition, Winter et al. found that damage to endothelial cells also causes increased t-PAIC concentrations in plasma (11). In previous studies, elevated t-PAIC plasma concentrations were observed in patients with both stroke (27) and myocardial infarction (28), and there was a correlation with PAI-1, reflecting impairment of the fibrinolytic activation pathway, which can be used to predict the occurrence of both diseases. However, the relationship between t-PAIC and cerebral infarction size is not clear. In our study, it was found that the plasma t-PAIC concentration in patients with MCI suggested that when MCI occurred in patients, there was not only excessive activation of the coagulation system and increased plasmin production, but also damage to the exogenous activation pathway of the fibrinolytic system, suggesting the necessity of initiating anticoagulant therapy in patients with MCI. In addition, the increase in plasma t-PAIC concentration also reflected that the vascular endothelial cell injury was more severe in patients with MCI, which could cause damage to the integrity of the blood–brain barrier, leading to adverse consequences such as malignant cerebral edema or cerebral hemorrhage.

TM is a transmembrane glycoprotein expressed in vascular endothelial cells and can be released into the blood circulation when the cell membrane is damaged by proteolytic enzymes. Increased concentration of plasma TM has been found to have some correlation with vascular endothelial injury in a variety of diseases with endothelial injury, and increased plasma-free TM is considered to be one of the specific indicators of endothelial cell injury (29). However, there is no consensus in the academic community on the correlation between plasma TM concentration and cerebral infarction, and some studies have shown that plasma TM concentration is increased in stroke patients compared with healthy individuals (30, 31); others have shown that the effect of plasma TM concentration on stroke patients is related to the history of stroke in stroke patients, and for those with a history of stroke, patients with higher plasma TM have a better prognosis after another stroke (32); however, so far there is no literature about the performance of TM in massive cerebral infarction. Through this study, we found that the mean concentration of TM plasma concentration was higher in patients with NMCI and fluctuated more in patients with MCI, but there was no significant statistical difference between the NMCI and MCI groups (*P* = 0.905), which may be affected due to the presence of different stroke subtypes. Cardioembolic stroke patients accounted for a greater proportion of patients with MCI, while patients with NMCI were mostly of the large artery atherosclerotic type. Previous studies have confirmed that plasma TM concentrations were higher in patients with acute stroke accompanied by large artery atherosclerosis (33), while the increase in plasma TM was not significant in patients with cardioembolic stroke (34).

## 5. Conclusion

The detection of PIC, TAT, t-PAIC, and TM can be used to comprehensively evaluate the vascular endothelial damage and the activation degree of the coagulation and fibrinolysis system. Meanwhile, it is easy to obtain the detection results, so these markers have diagnostic value in identifying patients with MCI and can be used as plasma markers for early detection of macrovascular occlusion. Their wide application will greatly improve the treatment efficiency of patients with macrovascular occlusion. For patients with plasma PIC ≥0.80 μg/ml, TAT ≥3.97 ng/ml or t-PAIC ≥ 9.85 ng/ml, we need to be alert to the possibility of MCI and transport them to advanced stroke centers that can perform thrombolysis or embolectomy as soon as possible, so as to save patients' lives and improve their prognosis.

## Data availability statement

The raw data supporting the conclusions of this article will be made available by the authors, without undue reservation.

## Ethics statement

The studies involving human participants were reviewed and approved by Human Ethics Review Committee of Shanxi Provincial People's Hospital. Written informed consent for participation was not required for this study in accordance with the national legislation and the institutional requirements.

## Author contributions

XZ: conceptualization, project administration, methodology, and writing–review and editing. SY: investigation, data curation, formal analysis, and writing–original draft. RL: writing–original draft. QD and JL: investigation and supervision. JM: investigation and data curation. LS: investigation. All authors contributed to the article and approved the submitted version.
